# Retigabine, a K_v_7.2/K_v_7.3-Channel Opener, Attenuates Drug-Induced Seizures in Knock-In Mice Harboring *Kcnq2* Mutations

**DOI:** 10.1371/journal.pone.0150095

**Published:** 2016-02-24

**Authors:** Yukiko Ihara, Yuko Tomonoh, Masanobu Deshimaru, Bo Zhang, Taku Uchida, Atsushi Ishii, Shinichi Hirose

**Affiliations:** 1 Department of Pediatrics, School of Medicine, Fukuoka University, Fukuoka, Japan; 2 Department of Chemistry, Faculty of Science, Fukuoka University, Fukuoka, Japan; 3 Department of Biochemistry, School of Medicine, Fukuoka University, Fukuoka, Japan; 4 Central Research Institute for the Molecular Pathomechanisms of Epilepsy, Fukuoka University, Fukuoka City, Japan; University of Modena and Reggio Emilia, ITALY

## Abstract

The hetero-tetrameric voltage-gated potassium channel K_v_7.2/K_v_7.3, which is encoded by *KCNQ2* and *KCNQ3*, plays an important role in limiting network excitability in the neonatal brain. K_v_7.2/K_v_7.3 dysfunction resulting from *KCNQ2* mutations predominantly causes self-limited or benign epilepsy in neonates, but also causes early onset epileptic encephalopathy. Retigabine (RTG), a K_v_7.2/ K_v_7.3-channel opener, seems to be a rational antiepileptic drug for epilepsies caused by *KCNQ2* mutations. We therefore evaluated the effects of RTG on seizures in two strains of knock-in mice harboring different *Kcnq2* mutations, in comparison to the effects of phenobarbital (PB), which is the first-line antiepileptic drug for seizures in neonates. The subjects were heterozygous knock-in mice (*Kcnq2*^Y284C/+^ and *Kcnq2*^A306T/+^) bearing the Y284C or A306T *Kcnq2* mutation, respectively, and their wild-type (WT) littermates, at 63–100 days of age. Seizures induced by intraperitoneal injection of kainic acid (KA, 12mg/kg) were recorded using a video-electroencephalography (EEG) monitoring system. Effects of RTG on KA-induced seizures of both strains of knock-in mice were assessed using seizure scores from a modified Racine’s scale and compared with those of PB. The number and total duration of spike bursts on EEG and behaviors monitored by video recording were also used to evaluate the effects of RTG and PB. Both *Kcnq2*^Y284C/+^ and *Kcnq2*^A306T/+^ mice showed significantly more KA-induced seizures than WT mice. RTG significantly attenuated KA-induced seizure activities in both *Kcnq2*^Y284C/+^ and *Kcnq2*^A306T/+^ mice, and more markedly than PB. This is the first reported evidence of RTG ameliorating KA-induced seizures in knock-in mice bearing mutations of *Kcnq2*, with more marked effects than those observed with PB. RTG or other K_v_7.2-channel openers may be considered as first-line antiepileptic treatments for epilepsies resulting from *KCNQ2* mutations.

## Introduction

K_v_7.2/K_v_7.3, a hetero-tetrameric voltage-gated potassium channel, consists of two types of subunits, which are encoded by *KCNQ2* and *KCNQ3*. K_v_7.2/K_v_7.3 is predominantly expressed in the hippocampus, neocortex, and the granular layer of the cerebellum [1–4] and generates the neuronal M-current, which stabilizes the membrane potential and controls neuronal excitability. K_v_7.2/K_v_7.3 thus plays an important role in limiting network excitability in the neonatal brain, where GABAergic action is depolarizing and excitatory [5].

Mutations in *KCNQ2* and *KCNQ3* are known to cause predominantly benign familial or non-familial neonatal epilepsy (BFNE or BNE) [2,6,7], both of which remit spontaneously in late infancy and thus are self-limited. However, a recent line of evidence shows that some *KCNQ2* mutations also cause early onset epileptic encephalopathies (EOEEs) or early infantile epileptic encephalopathies (EIEE), such as Ohtahara syndrome [8–12], which are associated with intractable seizures followed by profound psychomotor delay. In general, most individuals with BFNE or BNE have a benign course; however, some patients may have varying degrees of developmental delays and epilepsy recurring later in their life [11,12]. The development of rational therapy for epilepsies caused by dysfunctions resulting from mutated K_v_7.2/K_v_7.3 is thus urgently needed.

Retigabine (RTG), a K_v_7.2/K_v_7.3 opener, increases open channel probability and leads to hyperpolarization of the membrane potential. Hence, RTG may stabilize the resting membrane potential and suppress repetitive firing caused by *KCNQ2* mutations. Several *in vitro* electrophysiological studies on reconstituted K_v_7.2/K_v_7.3 have suggested that seizures caused by *KCNQ2* mutations might respond to RTG [13]. However, no study has evaluated the effects of RTG on seizures in genetically engineered animals harboring *Kcnq2* mutations, or compared these effects with the effects of phenobarbital (PB). At present, PB is the first-line and most commonly used anti-epileptic drug (AED) for neonatal seizures, including BFNE or EOEEs. Therefore, we here used knock-in mice bearing mutations in *Kcnq2*, the mouse orthologue of *KCNQ2*, to compare the effects of RTG on drug-induced seizures in the animals with those of PB.

## Materials and Methods

### Animal subjects

**Fig 1:**
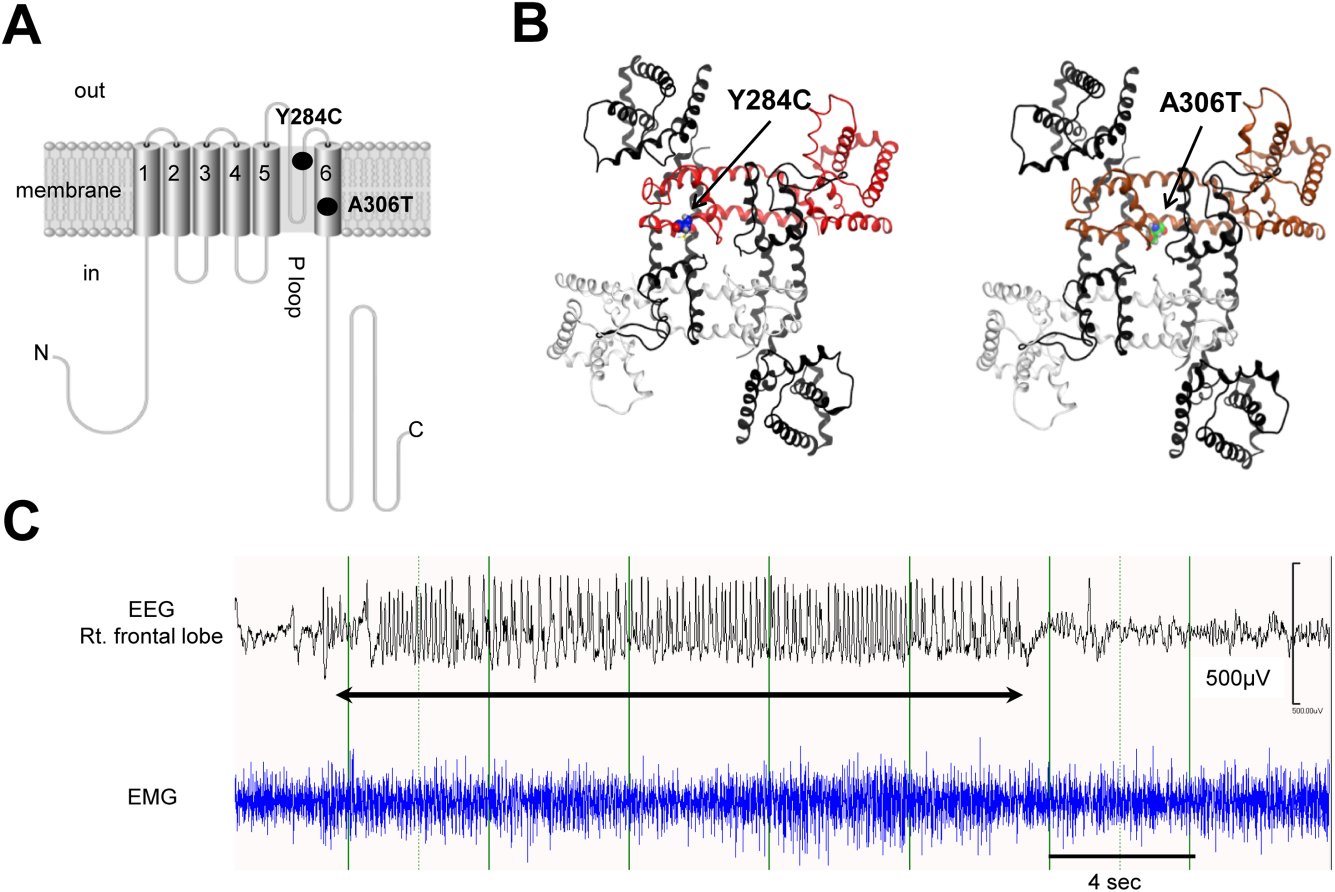
**A. Molecular structure of the KCNQ2 subunit.** The KCNQ2 subunit has six transmembrane domains (S1–S6), of which the 4^th^ transmembrane domain (S6) functions as a voltage sensor, whereas a loop between the 5^th^ and 6^th^ transmembrane domains (S5 and S6, respectively) forms an ion pore along with three other counterpart subunits. In general, the KCNQ2 and KCNQ3 subunits form a heterotetrametric potassium channel, i.e., K_v_7.2/K_v_7.3. The K_v_7.2/K_v_7.3 channel generates M-currents that control the subthreshold excitability of the membrane. p.Tyr284 and p.Ala306, where the mutations examined in this study (Y284C and A306T) are located, occur at the outer mouth and the inner lining (S6) of the channel pore, respectively. **B. Three dimensional images of the K**_**v**_**7.2/K**_**v**_**7.3 channels bearing Y284C and A306T.** The images indicate the outer mouth of the channel seen from an extracellular position. *Left*: The heterotetrametric K_v_7.2/K_v_7.3 channel consists of two KCNQ3 subunits (Black), one normal KCNQ2 subunit (White), and the mutant KCNQ2 subunit (Red) bearing the Y284C mutation. The cysteine residue introduced by the Y284C mutation, which is indicated with balls, is located at the outer mouth of the channel. *Right*: The heterotetrametric K_v_7.2/K_v_7.3 channel consists of two KCNQ3 subunits, one normal KCNQ2 subunit, and one mutant KCNQ2 subunit bearing the A306T mutation. The tyrosine residue introduced by the A306T mutation, which is indicated with balls, is located at the inner lining of the channel pore. **C. Spike burst.** Top: A spike burst recorded during a kainic acid challenge test on an electroencephalogram (EEG) is indicated with a double sided arrow Bottom: A simultaneous electromyogram (EMG) recording. A spike burst is a cluster of high-amplitude and high frequency spikes, each of which lasts a few seconds to several minutes.

Two strains of heterozygous knock-in mice, *Kcnq2*^Y284C/+^ and *Kcnq2*^A306T/+^, which harbor heterozygous Y284C or A306T *Kcnq2* mutations, respectively, were produced using the “kick-in” system as described elsewhere [14]. *Kcnq2*^Y284C/+^ and *Kcnq2*^A306T/+^ mice used in this study were congenic strains produced by more than 10 repeated backcross to C57BL/6J strain mice. Heterozygous mutations of Y284C and A306T have previously been identified in *KCNQ2* in individuals with BFNE [15]. Both Y284C and A306T mutations are known to cause the BFNE phenotype, but phenotypic difference in patients has not been reported. Y284C is located in the loop that forms the ion pore of the channel, whereas A306T is located in transmembrane segment 6 (Fig 1A). Y284C and A306T are located at the outer mouth and the inner lining of the channel pore, respectively (Fig 1B). In this study, we used both *Kcnq2*^Y284C/+^ and *Kcnq2*^A306T/+^ mice to strengthen the results to evaluate potential differences, if any, between the two mutants.

The “kick-in” system that we previously developed is a modified knock-in procedure making use of Cre/mutant *lox* technology [14]. The system allows rapid production of multiple strains of knock-in mice bearing different mutations in a given portion of target genes. *Kcnq2*^Y284C/+^ and *Kcnq2*^A306T/+^ mice have been found to exhibit occasional spontaneous seizures. In contrast to these sparse spontaneous seizures, seizures can be more readily induced by proconvulsants, such as pentylenetetrazol, in both *Kcnq2*^Y284C/+^ and *Kcnq2*^A306T/+^ mice compared to their wild-type (WT) littermates [14].

Mice were housed at 23 ± 2°C with 12 h light−12 h dark cycle (light on 7:00 to 19:00) and were given free access to commercial chow and tap water. Mice used for all experiments were 63–100 days of age (WT mice, n = 13; *Kcnq2*^Y284C/+^ mice, n = 40; *Kcnq2*^A306T/+^ mice, n = 33). To conduct all experiments blindly, genotyping to distinguish knock-in mice from their WT littermates was performed after each experiment, according to methods described elsewhere [14].

### Ethics and animal rights protection

The experimental protocols were approved by the Committee for Animal Care and Use of Fukuoka University (Approval number: 294) and all experiments were conducted in compliance with the animal experimentation guidelines “Basic policies on animal experimentation" issued by the Ministry of Education, Sports, Science, and Technology (MEXT) and the Ministry of Health, Labor, and Welfare (MHLW) in Japan. All surgery was performed under sodium pentobarbital anesthesia, and efforts were made to minimize stress to the animals. Because all drug-induced seizures were monitored using a video-electroencephalography (EEG) monitoring system, we were able to observe in real time to determine whether mice were overstressed during seizures. During the course of the study, one *Kcnq2*^A306T/+^ mouse died after recurrent generalized tonic-clonic seizures and wild jumping approximately 30 minutes after kainic acid (KA) injection. The cause of death is unknown, but may have involved asphyxia or asystole. After the experiments, the mice were euthanized using sodium pentobarbital to minimize suffering.

### Structural modeling and docking of the K_v_7.2/K_v_7.3 channel

The stereoscopic protein structure model of the human K_v_7.2/K_v_7.3 channel transmembrane domain was remodeled from a PDB file provided by Prof. Bernard Attali (Department of Physiology & Pharmacology, Sackler Faculty of Medicine, Tel Aviv University, Israel) [16], which is a modified chimeric version containing the rat K_V_2.1 paddle (PDB entry 2R9R). Tetrameric reconstruction and energy minimization were calculated using *Molecular Operating Environment (MOE)*, 2013.08 (Chemical Computing Group Inc., 1010 Sherbooke St. West, Suite #910, Montreal, QC, Canada, H3A2R7, 2014).

### EEG with video monitoring

EEGs of mice were recorded with video monitoring as previously described [14]. In brief, mice were anesthetized with sodium pentobarbital (50 mg/kg body weight, i.p.), and bipolar stainless steel wire electrodes were implanted into the right forehead (2.0 mm anterior to the bregma, 1.5 mm lateral from the midline) and over the right hippocampus (2.0 mm posterior to the bregma, 1.5 mm lateral from the midline). Two electromyogram (EMG) wires were placed in the neck, between the muscle and skin. Digital EEG and video monitoring were performed in the cage for 1 week after the operation (KISSEI COMTEC, Sleep sign version 2, vital recorder, video option, Matsumoto, Japan).

A spike burst is a cluster of high-amplitude and high frequency spikes, each of which lasts a few seconds to several minutes on EEG during KA challenge tests [17] (Fig 1C). The total number and duration of the spike bursts observed for 120 min after KA injection in *Kcnq2*^Y284C/+^ and *Kcnq2*^A306T/+^ mice and their WT littermates were compared.

### Drug-induced seizures

To induce seizures, mice received 12 mg/kg body weight KA (Sigma, St Louis, MO, USA) intraperitoneally 30 min after they had acclimated, as previously reported [18]. KA stocks, dissolved in water, were stored at -20°C; aliquots were diluted in normal saline solution immediately before use. The final concentration of KA used for injection was 1.2 mg/ml.

**Fig 2:**
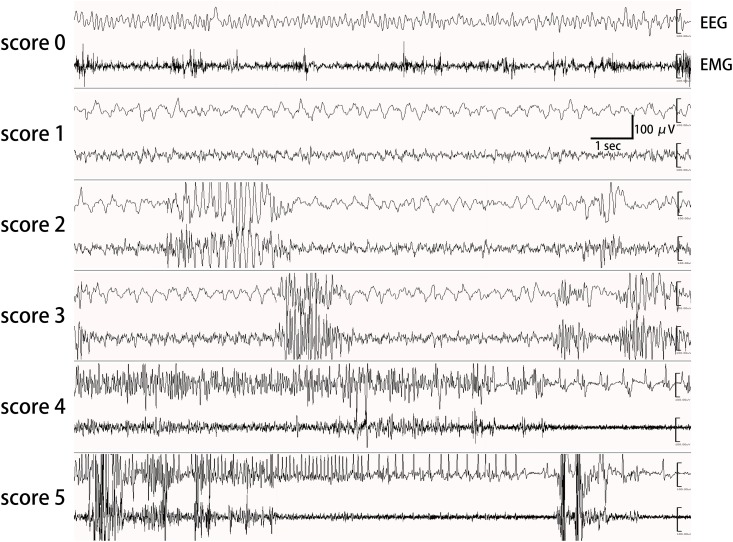
Representative electroencephalograms at each score of Modified Racine’s scale. Electroencephalogram (EEG) and electromyogram (EMG) were obtained as described in Method. Each panel shows a representative EEG recording at Score 0 to 5 of a Modified Racine’s scale [19]. Spikes and sharp waves were considered part of seizure activities only when they were associated with abnormal behaviors which were confirmed on video monitoring and electromyogram. A spike burst is defined as a cluster of high-amplitude and high frequency spikes, each of which lasts a few seconds to several minutes on EEG during kainic acid challenge tests [17].

The seizures and behaviors were recorded continuously for more than 120 min with EEGs and video monitoring after drug injection. Seizures were scored based on behavior and EEG findings using a modified Racine’s scale [19] (Table 1) (Fig 2). Spikes and sharp waves were considered to represent seizure activity only when they were associated with abnormal behaviors that were confirmed on video monitoring and EMG. We evaluated seizures and the effects of AEDs using this scoring system and also based on the number and total duration of spike bursts on EEG for 120 min after KA injection.

**Table 1:** Modified Racine’s scale.

Score	Behavioral stage	EEG findings
**0**	No change in behavior	Baseline
**1**	Sudden behavioral arrest, motionless staring (with orofacial automatism)	High amplitude activity/slow waves
**2**	Head nodding	Spikes, sharp waves
**3**	Forelimb clonus with lordotic posture	Spikes or poly spikes, sharp waves
**4**	Forelimb clonus, with rearing and falling	Spike bursts/spike and wave discharges
**5**	Generalized tonic-clonic activity with loss of postural tone, often resulting in death, wild jumping	Spike bursts/spike and wave discharges

### Anti-epileptic drugs

RTG dihydrochloride (Toronto Research Chemicals, Toronto, Canada) and PB sodium salt (WAKO, Osaka, Japan) were dissolved in physiological saline. All drugs were administered intraperitoneally 30 min before KA injection. Two doses of each AED (5 mg/kg and 15 mg/kg) were given for *Kcnq2*^Y284C/+^ and *Kcnq2*^A306T/+^ mice. Regardless of the dose, the injection volume was adjusted to 10 ml/kg bodyweight. We evaluated seizure scores and EEG findings as described above.

### Statistical data analyses

Statistical data analyses were performed using the SAS (Statistical Analysis System) Software Package (Ver. 9.4, SAS Institute Inc., Cary, NC, USA) at Fukuoka University. The association of heterozygous *Kcnq2* mutations with the seizure score, which is an ordinal measure, was examined using an exact test for ordered differences [20]. Differences in the distribution of the seizure score among WT, *Kcnq2*^Y284C/+^, and *Kcnq2* ^A306T/+^ mice or among mice administered vehicle, PB, or RTG were examined using the Jonckheere−Terpstra test [20] and the logistic regression analyses [21]. The number of spike bursts and the duration of spike bursts were compared among different types of mice, or mice treated with different types of drugs using Poisson regression [20]. Additional analyses were performed for overdispersion in Poisson regression [22]. Negative binomial model with a linear variance function (p = 1) was used to account for overdispersion when exists [22,23]. The t-statistics of dispersion parameter, labeled as “_Alpha” in the COUNTREG procedure, was used to assess the significance of overdispersion [22,23]. The significance level was considered to be less than 0.05, unless indicated otherwise.

## Results

### Higher sensitivity to KA-induced seizure in *Kcnq2* mutant mice

**Fig 3:**
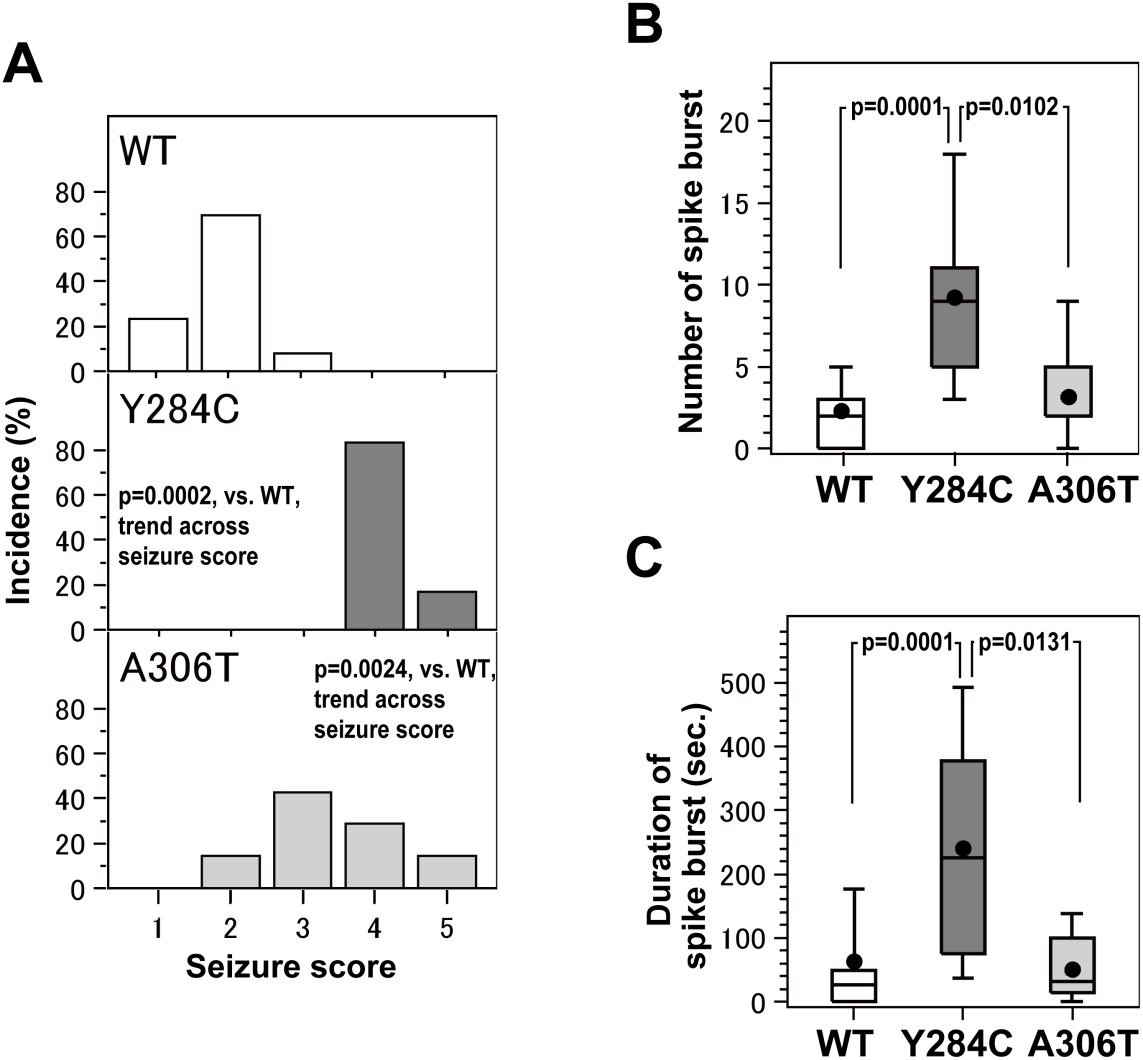
Relationship between heterozygous *Kcnq2* mutations and seizures. **A.** The distribution of seizure scores in WT mice (n = 13) (upper panel, in white color), *Kcnq2*^Y284C/+^ mice (n = 6) (middle panel, in gray color), and *Kcnq2* ^A306T /+^ mice (n = 7) (lower panel, in light gray color). Differences in the distribution of seizure scores among and between groups were examined by the logistic regression analysis, as described in the Methods. **B** and **C**. Box-and-whisker plots showing the mean (●), median (middle bar in the rectangle), and 10^th^ (bottom bar), 25^th^ (bottom of rectangle), 75^th^ (top of rectangle), and 90^th^ (top bar) percentiles of the number (B) and duration (C) of spike bursts in WT (n = 13) (in white color), *Kcnq2*^Y284C/+^ (n = 6) (in gray color), and *Kcnq2* ^A306T /+^ (n = 7) mice (in light gray color). The number and duration of spike bursts were compared among and between groups by using Negative binomial model, as described in the Methods. Raw data for bar and box plots are included in S1 File.

Both *Kcnq2*^Y284C/+^ and *Kcnq2*^A306T/+^ mice showed significantly more KA-induced seizures, with a higher seizure score, than WT mice (Fig 3A). In WT mice, no KA-induced seizure with a score of > 3 was observed. In contrast, in *Kcnq2*^Y284C/+^ mice, all scores of KA-induced seizures were ≥ 4. Similarly, KA-induced seizures in *Kcnq2*^A306T/+^ mice had higher scores than those observed in WT mice (Fig 3A). The differences in the distribution of the seizure score between the mutant and WT mice and between the two strains of mutant mice were statistically significant (*p* = 0.0002, *Kcnq2*^Y284C/+^ vs. WT; *p* = 0.0024, *Kcnq2*^A306T/+^ vs. WT; *p* = 0.0982, *Kcnq2*^Y284C/+^ vs. *Kcnq2*^A306T/+^), as assessed by the logistic regression analysis. These results indicate that *Kcnq2*^Y284C/+^ and *Kcnq2*^A306T/+^ mice have significantly higher sensitivity to KA-induced seizures than WT mice, and *Kcnq2*^Y284C/+^ mice have higher sensitivity to KA-induced seizures than *Kcnq2*^A306T/+^ mice.

In accordance with this higher seizure sensitivity to KA, *Kcnq2*^Y284C/+^ mice exhibited frequent and prolonged spike bursts during KA challenges (Fig 3B and 3C). *Kcnq2*^Y284C/+^ mice had significantly more and longer spike bursts than WT mice (*p* = 0.0001, Fig 3B and *p* = 0.0001, Fig 3C), as assessed by using Negative binomial model. Similarly, *Kcnq2*^A306T/+^ mice showed a tendency to have more and longer spike bursts than WT mice, although the difference was not statistically significant (*p* = 0.3357 and *p* = 0.2398). Furthermore, *Kcnq2*^Y284C/+^ mice had significantly more and longer spike bursts than *Kcnq2*^A306T/+^ mice (*p* = 0.0102, Fig 3B and *p* = 0.0131, Fig 3C), as assessed by using Negative binomial model. Thus, compared to WT and *Kcnq2*^A306T/+^ mice, *Kcnq2*^Y284C/+^ mice exhibited more frequent and prolonged spike bursts during KA challenges.

### RTG was superior to PB in ameliorating KA-induced seizures in *Kcnq2*^Y284C/+^ mice

**Fig 4:**
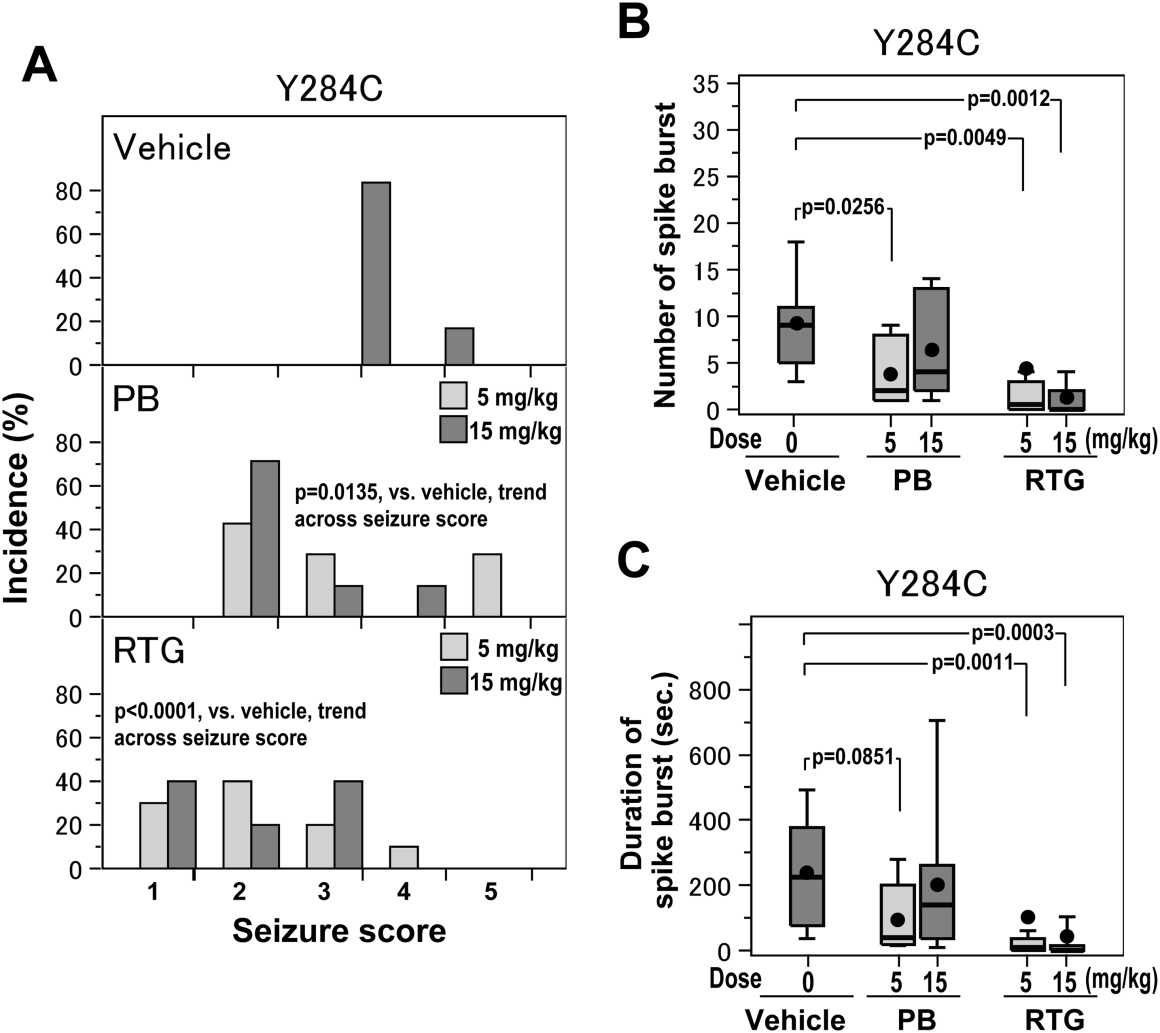
Effects of phenobarbital (PB) and retigabine (RTG) at low and high doses on seizures in *Kcnq2*^Y284C/+^ mice. **A.** Distribution of seizure scores in *Kcnq2*^Y284C/+^ mice administered vehicle (n = 6) (upper panel, in gray color), PB at doses of 5 mg/kg (n = 7) and 15 mg/kg (n = 7) (middle panel, in light gray and gray color, respectively), or RTG at doses of 5 mg/kg (n = 10) and 15 mg/kg (n = 10) (lower panel, in light gray and gray color, respectively). Between and within drug differences in the distribution of seizure scores were examined by the Jonckheere−Terpstra test, as described in the Methods. **B and C.** Box-and-whisker plots showing the mean (●), median (middle bar in the rectangle), and 10^th^ (bottom bar), 25^th^ (bottom of rectangle), 75^th^ (top of rectangle), and 90^th^ (top bar) percentiles of the number (B) and duration (C) of spike bursts in *Kcnq2*^Y284C/+^ mice administered vehicle (n = 6), PB at doses of 5 mg/kg (n = 7) and 15 mg/kg (n = 7), or RTG at doses of 5 mg/kg (n = 10) and 15 mg/kg (n = 10). PB and RTG at doses of 5 mg/kg are shown in light gray color. Between and within drug differences in terms of the number and duration of spike bursts were examined using Negative binomial model, as described in the Methods. Raw data for bar and box plots are included in S1 File.

Administration of PB or RTG (5 mg/kg and 15 mg/kg) prior to KA challenges reduced the incidence of KA-induced seizures in the *Kcnq2*^Y284C/+^ mice (Fig 4A). The trend across seizure scores indicated significantly better effects for pretreatment with PB (at doses of 5 and 15 mg/kg) or RTG (at doses of 5 and 15 mg/kg) than with vehicle (*p* = 0.0135, PB vs. vehicle; *p* < 0.0001, RTG vs. vehicle, Fig 4A), as assessed by the Jonckheere−Terpstra test. The preventative effect of RTG was greater than that of PB (*p* = 0.0766), after adjusting for dose by the logistic regression analysis.

Next, we assessed the dose-dependency of the effects of PB and RTG on seizure scores (Fig 4A). The preventative effect of 15 mg/kg PB on KA-induced seizures was statistically significant (*p* = 0.0041, 15 mg/kg PB vs. vehicle), although the effect of 5 mg/kg of PB was not statistically significant (*p* = 0.1404, 5 mg/kg PB vs. vehicle), as assessed by the Jonckheere−Terpstra test. There was no significant difference between the effects of 5 mg/kg and 15 mg/kg PB in *Kcnq2*^Y284C/+^ mice (*p* = 0.2884, 15 mg/kg vs. 5 mg/kg PB, Fig 4A).

In contrast to PB, RTG demonstrated significant preventative effects for KA-induced seizures at both doses (*p* = 0.0012, 5 mg/kg RTG vs. vehicle; *p* = 0.0002, 15 mg/kg RTG vs. vehicle), as assessed by the Jonckheere−Terpstra test. There was no significant difference in the preventative effects between the two doses of RTG (*p* = 0.8829, 15 mg/kg vs. 5 mg/kg RTG, Fig 4A). Thus, pretreatment with RTG achieved a significant preventative effect for KA-induced seizures even at a low dose (5 mg/kg) in *Kcnq2*^Y284C/+^ mice.

The difference between the effects of PB and RTG in these mice became evident when they were compared in terms of spike bursts (Fig 4B and 4C). Thus, RTG was superior to PB in ameliorating KA-induced seizures, although both PB and RTG were effective in reducing the number and total duration of spike bursts during KA challenges in *Kcnq2*^Y284C/+^ mice.

Both PB and RTG reduced the number of spike bursts (Fig 4B). PB at a dose of 5 mg/kg produced a significant reduction of the number of spike bursts (*p* = 0.0256, 5 mg/kg PB vs. vehicle, Fig 4B), whereas 15 mg/kg PB did not have a statistically significant effect (*p* = 0.2171, 15 mg/kg PB vs. vehicle), as assessed by using Negative binomial model. There was no significant difference between these two doses of PB in terms of the number of spike bursts. RTG, in contrast, reduced the number of spike bursts significantly at both 5 mg/kg and 15 mg/kg doses (*p* = 0.0049, 5 mg/kg RTG vs. vehicle; *p* = 0.0012, 15 mg/kg RTG vs. vehicle, Fig 4B), as assessed by using Negative binomial model. This effect of RTG was not significantly dose-dependent (*p* = 0.5358, 15 mg/kg vs. 5 mg/kg RTG). The effects of RTG on the number of spike bursts were significantly better than those of PB in *Kcnq2*^Y284C/+^ after adjusting for dose (*p* = 0.0026).

In accordance with the effects on the number of spike bursts, PB and RTG were effective in shortening the total duration of spike bursts, and the effects of RTG were more marked than that of PB (Fig 4C). PB, at a dose of 5 mg/kg, reduced the total duration of spike bursts (*p* = 0.0851, 5 mg/kg PB vs. vehicle, Fig 4C), as assessed by using Negative binomial model. In contrast, RTG shortened the total duration of spike bursts significantly at both 5 mg/kg and 15 mg/kg doses (*p* = 0.0011, 5 mg/kg RTG vs. vehicle; and *p* = 0.0003, 15 mg/kg RTG vs. vehicle, Fig 4C), as assessed by using Negative binomial model. This effect of RTG was not significantly dose-dependent (*p* = 0.5815, 15 mg/kg vs. 5 mg/kg RTG). The effects of RTG on shortening the total duration of spike bursts were significantly better than those of PB after adjusting for dose (*p* < 0.0001).

### RTG was superior to PB in ameliorating KA-induced seizures in *Kcnq2*^A306T/+^ mice

**Fig 5:**
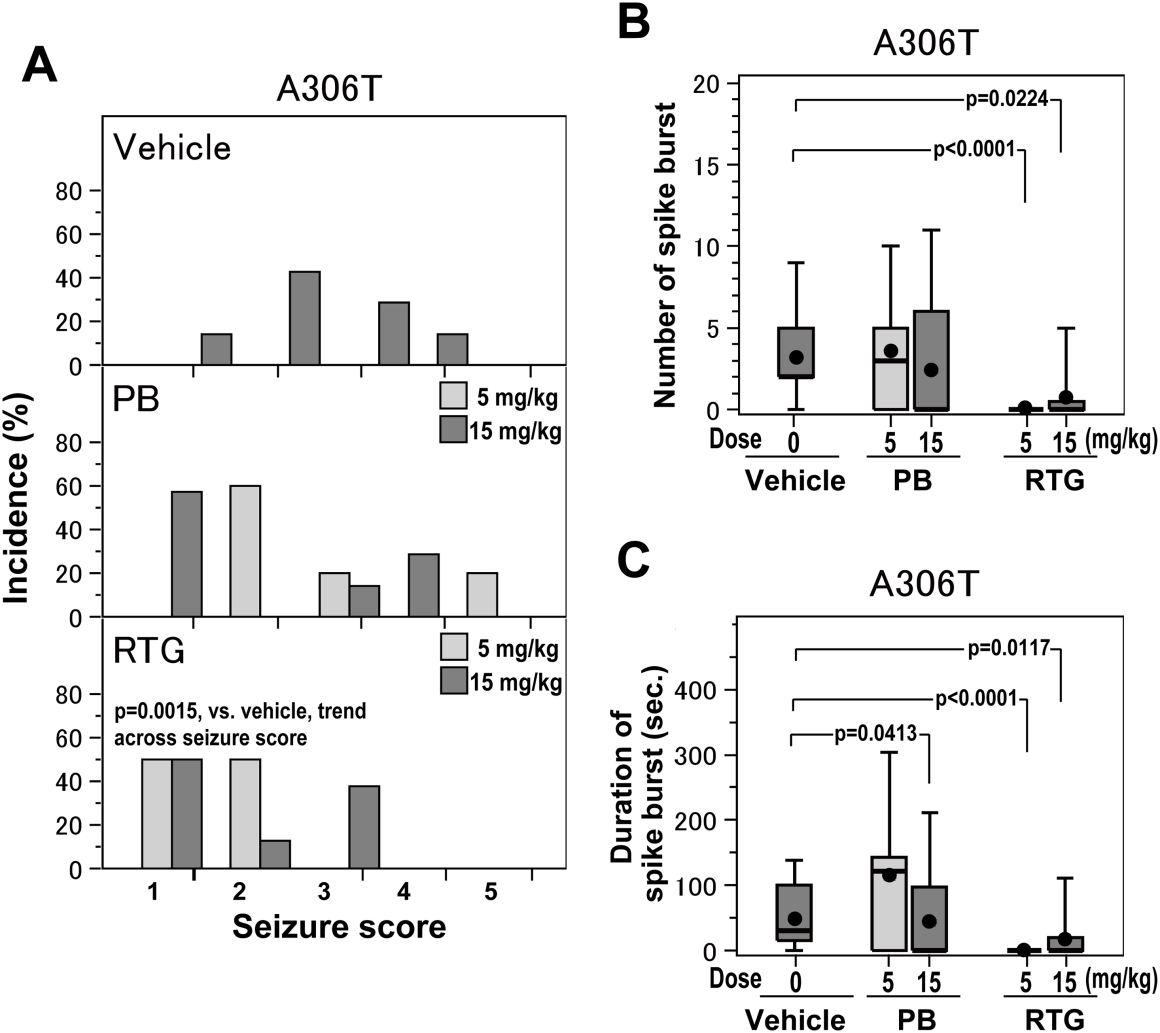
The effects of phenobarbital (PB) and retigabine (RTG) at low and high doses on seizure in *Kcnq2*^A306T/+^ mice. **A.** Distribution of seizure scores in *Kcnq2*^A306T/+^ mice administered vehicle (n = 7) (upper panel, in gray color), PB at doses of 5 mg/kg (n = 5) and 15 mg/kg (n = 7) (middle panel, in light gray and gray color, respectively), or RTG at doses of 5 mg/kg (n = 6) and 15 mg/kg (n = 8) (lower panel, in light gray and gray color, respectively). Between and within drug differences in the distribution of seizure scores were examined by the Jonckheere−Terpstra test, as described in the Methods. **B and C.** Box-and-whisker plots showing the mean (●), median (middle bar in the rectangle), and 10^th^ (bottom bar), 25^th^ (bottom of rectangle), 75^th^ (top of rectangle), and 90^th^ (top bar) percentiles of the number (B) and duration (C) of spike bursts in *Kcnq2*^A306T/+^ mice administered vehicle (n = 7), PB at doses of 5 mg/kg (n = 5) and 15 mg/kg (n = 6), or RTG at doses of 5 mg/kg (n = 6) and 15 mg/kg (n = 8). PB and RTG at doses of 5 mg/kg are shown in light gray color. Between and within drug differences in terms of the number and duration of spike bursts were examined using Negative binomial model, as described in the Methods. Raw data for bar and box plots are included in S1 File.

The effects of PB and RTG were also evaluated and compared in heterozygous *Kcnq2*^A306T/+^ mice (Fig 5). Administration of PB and RTG (5 mg/kg and 15 mg/kg) prior to KA challenges reduced the incidence of KA-induced seizures in *Kcnq2*^A306T/+^mice (Fig 5A). The trend across seizure scores indicated significantly better effects of pretreatment with RTG (at doses of 5 and 15 mg/kg) than with vehicle (*p* = 0.0015, RTG vs. vehicle, Fig 5A), although the preventive effects of PB (at doses of 5 and 15 mg/kg) on KA-induced seizures was not statistically significant (*p* = 0.1144, PB vs. vehicle), as assessed by the Jonckheere−Terpstra test. Furthermore, RTG had better preventive effects on KA-induced seizures than PB in *Kcnq2*^A306T/+^ mice, although the difference did not reach statistical significance (*p* = 0.1857), after adjusting for dose by the logistic regression analysis.

Next, we assessed the dose-dependency of the effects of RTG on KA-induced seizure score in *Kcnq2*^A306T/+^ mice (Fig 5A). Both 5 mg/kg RTG and 15 mg/kg RTG demonstrated significant preventative effects on KA-induced seizures (*p* = 0.0029, 5 mg/kg RTG vs. vehicle; *p* = 0.0165, 15 mg/kg RTG vs. vehicle), but the effect of RTG was not significantly dose-dependent (*p* = 0.6084, 15 mg/kg vs. 5 mg/kg RTG, Fig 5A), as assessed by the Jonckheere−Terpstra test. Thus, similar to *Kcnq2*^Y284C/+^ mice, pretreatment with RTG achieved a significant preventative effect even at a low dose in *Kcnq2*^A306T/+^ mice.

The difference between PB and RTG became more evident when they were compared in terms of their effects on both the number and total duration of spike bursts during KA challenges. Thus, RTG was superior to PB in ameliorating KA-induced seizures in terms of reducing the number and total duration of spike bursts during KA challenges (Fig 5B and 5C).

RTG demonstrated the ability to significantly reduce the number of spike bursts during KA challenges at both 5 mg/kg and 15 mg/kg doses (*p* < 0.0001, 5 mg/kg RTG vs. vehicle; *p* = 0.0224, 15 mg/kg RTG vs. vehicle; Fig 5B), whereas the effects of PB at 5 mg/kg and 15 mg/kg doses on the number of spike bursts were not statistically significant (*p* = 0.7222, 5 mg/kg PB vs. vehicle; *p* = 0.1174, 15 mg/kg PB vs. vehicle), as assessed by using Negative binomial model. The effects of RTG in terms of the number of spike bursts were significantly better than those of PB in *Kcnq2*^A306T/+^ mice after adjusting for dose (*p* = 0.0603).

In accordance with the effects on the number of spike bursts, PB and RTG were effective in shortening the total duration of spike bursts, and the effect of RTG was more marked than that of PB (Fig 5C). The effect of PB on shortening the total duration of spike bursts was significant at a dose of 15 mg/kg but not at 5 mg/kg (*p* = 0.0413, 15 mg/kg PB vs. vehicle; *p* = 0.5787, 5 mg/kg PB vs. vehicle, Fig 5C), as assessed by using Negative binomial model. In contrast, RTG demonstrated a significant effect at both 5 mg/kg and 15 mg/kg doses (*p* < 0.0001, 5 mg/kg RTG vs. vehicle; *p* = 0.0117, 15 mg/kg RTG vs. vehicle, Fig 5C) on shortening the total duration of spike bursts, as assessed by using Negative binomial model. The difference between RTG and PB in terms of the total duration of spike bursts in *Kcnq2*^A306T/+^ mice was also statistically significant after adjusting for doses (*p* = 0.0740).

## Discussion

In the present study, both *Kcnq2*^Y284C/+^ and *Kcnq2*^A306T/+^ heterozygous mutant mice showed significantly higher sensitivity to KA-induced seizures than WT mice. Accordingly, both mutant mice exhibited more frequent and prolonged spike bursts during KA challenges. RTG, a pan K_v_7.2-K_v_7.5-channel opener [24–28], suppressed these KA-induced seizure activities more effectively than PB, which is currently the first-line AED for neonatal seizures, including BFNE, resulting from *KCNQ2* mutations.

### *Kcnq2*^Y284C/+^ and *Kcnq2*^A306T/+^ mice are prone to KA induced seizures

We previously showed high sensitivity to proconvulsive treatment in *Kcnq2*^Y284C/+^ and *Kcnq2*^A306T/+^ mice, using pentylenetetrazole (PTZ) [14]. Similar findings were reported in early studies with *Szt1* mice, a spontaneous mutant mouse strain that harbors a microdeletion affecting the C-terminus of mouse *Kcnq2* and also its adjacent two genes, *Chnra4* and *Arfgap-1* [29–33]. Heterozygous *Kcnq*2 knock-out mice have also demonstrated hypersensitivity to PTZ [34]. Furthermore, a high sensitivity to proconvulsive treatments has been observed in BFNE-mutation knock-in mice that were independently generated by another group. These mutations included the A306T mutation in *Kcnq2* and the G311V mutation in *Kcnq3*, which is an ortholog of a *KCNQ3* mutation found in human BFNE [35,36]. Similar to our *Kcnq2*^Y284C/+^ and *Kcnq2*^A306T/+^ mice, these heterozygous mutant mice did not show spontaneous seizures, although their homozygotes did. The *Kcnq2*^A306T/+^ and *Kcnq3*^G311V/+^ mice had significantly higher sensitivity to electroconvulsive seizure and kindling acquisition [35,36]. Interestingly, they demonstrated genotype-related differences in sensitivity. In agreement with this indication, our present study found that *Kcnq2*^Y284C/+^ mice were more prone to KA-induced seizures than *Kcnq2*^A306T/+^ mice in terms of the frequency and duration of seizure bursts upon EEG, suggesting that the BFNE mutations vary in sensitivity.

Thus, the hypersensitivity to proconvulsants observed in *Kcnq2*-deficient mice and the resulting impairment of M-currents hamper the stabilization of the resting membrane potential and subthreshold levels of membrane excitability [37]. Therefore, these findings in mouse models provide compelling evidence for the crucial role of M-currents in controlling neuronal excitability [5], supporting findings from electrophysiological studies on reconstituted channels *in vitro* [38–44].

### RTG significantly attenuates KA-induces seizures in *Kcnq2*^Y284C/+^ and *Kcnq2*^A306T/+^ mice

In both *Kcnq2*^Y284C/+^ and *Kcnq2*^A306T/+^ mice, RTG, an opener of K_v_7 channels, was more effective in preventing KA-induced seizures than PB, not only in terms of the incidence of seizures, but also in terms of suppressing the spike bursts on EEG.

To our knowledge, this is the first application and comparison of RTG with other AEDs in genetically engineered animal models bearing *Kcnq2* BFNE mutations. The effects of another potassium channel opener, flupirtine, which is an analogue of RTG, were previously evaluated and compared with PB, albeit in experimental seizure models involving WT rodents. Flupirtine has a better effect on experimental seizures than PB [45].

In addition, RTG has been used in *Szt1* mice, but was found less effective in reducing electroconvulsions in *Szt1* mice than in their WT controls [30]. However, its effect was not compared with those of other AEDs in this model [30]. Therefore, the present study, for the first time, has provided evidence that RTG would provide better prevention of seizure activity in the presence of genetically impaired K_v_7.2.

Our findings also support *in vitro* electrophysiological findings that RTG ameliorates the dysfunction of K_v_7.2 caused by *KCNQ2* mutations, obtained either from reconstituted channels in *Xenopus laevis* oocytes [44,46] or brain slice patch clamping in mice bearing a *KCNQ2* microdeletion [37,47–49]. Interestingly, a report of a voltage clamping assay assessing the effect of RTG on reconstituted K_v_7.2 in *Xenopus laevis* oocytes revealed that the effect of RTG on lowering action potential in WT K_v_7.2 was significantly greater than that on K_v_7.2 harboring EOEE mutations [44]. BFNE-related *KCNQ2* mutations cause haploinsufficiency, while EOEE mutations cause a dominant-negative effect. Hence, given that K_v_7.2/K_v_7.3 is a hetero-tetramer, RTG may exert its function in both *Kcnq2*^Y284C/+^ and *Kcnq2*^A306T/+^ mice by ameliorating the pore region, which consists of two KCNQ3 subunits and one normal KCNQ2 subunit derived from WT alleles (Fig 1B).

### RTG is more effective than PB against KA-induced seizures in *Kcnq2*^Y284C/+^ and *Kcnq2*^A306T/+^ mice

PB is widely used as the first-line AED for neonatal seizures, including BFNE resulting from *KCNQ2* mutations. PB exerts its pharmacological function mainly as a GABA_A_ receptor agonist, but also as an antagonist of the AMPA/kainate−glutamate receptor, inhibiting glutamate release, which is controlled by the P/Q-type calcium channel [50,51]. The NMDA and AMPA subtypes of glutamate receptors are highly expressed between the first and second postnatal weeks in rats and in the neonatal period in humans [52,53]. It is therefore believed that PB is useful for treating neonatal seizures because the additional reduction of glutamate receptors may reduce the severity of neonatal seizures [51].

In the neonatal brain, however, the environment and function of neurotransmitters are different from those in the mature brain. For example, GABAergic action is depolarizing and excitatory in the neonatal brain because of the higher intracellular chloride concentration caused by the predominant expression of *NKCC1* over that of *KCC2* [5,54–56]. The composition of the subunits of GABA_A_ receptors also changes during brain development [57,58]. Thus, the α4 subunits of GABA receptors, which render the receptor less sensitive to benzodiazepines, are relatively overexpressed at developmental stages compared with GABA_A_ receptors in the adult brains that consist predominantly of the α1 subunit [57,59,60]. Some AEDs, such as PB and benzodiazepines, function as agonists to GABA_A_ receptors and may be less effective in neonates than in adults because of the characteristics of the GABA_A_ receptor in the neonatal brain [61,62]. Consequently, intractable neonatal seizures, for which PB does not work well, are clinically encountered.

In contrast, RTG exerts its antiepileptic effect by opening K_v_7 channels, including K_v_7.2 and K_v_7.3. RTG binds to a hydrophobic pocket in the ion pore between transmembrane segments S5 and S6 (Fig 1A and 1B), opening the pore and increasing the membrane potassium conductance of neurons [63]. The increment of potassium currents reduces the generation of action potentials. Therefore, RTG can both suppress the hyperexcitability of neurons via potassium channels and function as an AED [63]. In addition, K_v_7.2/K_v_7.3 channels are predominantly expressed in the axon initial segment of neurons, which is a region crucial for controlling neuronal excitability [37,64,65]. Furthermore, K_v_7.2 and K_v_7.3 appear to be particularly important for the neonatal brain, as they are highly expressed from late fetal life to early infancy [66,67]. Although RTG is known to cause positive allosteric modulation of GABA_A_ receptors, this effect is observed only at high concentrations, and no significant interaction with glutamate receptors has been observed [24,68]. Therefore, RTG is considered a rational AED for use in neonatal seizures, specifically for those resulting from K_v_7.2 or K_v_7.3 dysfunction.

### Potassium channel openers in neonatal seizures

RTG has been approved by the FDA as an add-on therapy for partial-onset seizures [69]. However, clinical application of RTG has recently been limited because of rising concerns regarding its adverse effects, such as blue skin discoloration and eye abnormalities resulting from pigment changes in the retina [70,71]. Nevertheless, several lines of evidence, including our findings on the efficacy of RTG for reducing seizures in *Kcnq2* mutant mice, warrant further investigation of the therapeutic potential of RTG and related potassium-channel openers [37,64,65]. In addition, mutations of *KCNQ2* are known to cause not only benign epilepsy, but also malignant phenotypes, e.g., EOEE and Ohtahara syndrome, for which treatments based on an understanding of the underlying pathomechanisms are urgently required [8–11]. RTG has been shown to be effective for seizures resulting from *KCNQ*2 mutations with a dominant-negative effect [44,72,73], which is suspected to be an underlying mechanism of some cases of EOEE. In addition, a new potassium-channel opener with fewer side effects was recently developed and has been shown to be effective for epilepsy [74].

Taken together, these previous findings and our present study suggest high potential of RTG in treating epilepsy resulting from *KCNQ2* mutations. However, there are limitations in applying the results of the present study directly to human BFNE or EOEE. Seizures in the mice were induced by KA and not spontaneous, and the age of the mice used does not necessarily correspond to neonates. Therefore, further study of potassium-channel openers should provide insights into the treatment of neonatal seizures in the near future.

## Conclusions

We have here provided the first evidence that RTG, a K_v_7 potassium-channel opener, ameliorates KA-induced seizures in knock-in mice bearing mutations of *Kcnq2*, the orthologue of *KCNQ2* that encodes K_v_7.2 channels in humans. Furthermore, the effect of RTG is superior to that of PB, the accepted first-line AED for neonatal seizures. Given the efficacy of RTG in animal models of neonatal epilepsy caused by *KCNQ2* mutations, potassium channel openers should be considered as a therapeutic option for BFNE/BNE and perhaps even for EOEE.

## Supporting Information

S1 File Dataset for bar and box plots. (XLSX)
